# Descriptive Analysis of Extubations Performed in an Emergency Department-based Intensive Care Unit

**DOI:** 10.5811/westjem.2020.4.47475

**Published:** 2020-04-24

**Authors:** Nathan L. Haas, Patrick Larabell, William Schaeffer, Victoria Hoch, Miguel Arribas, Amanda C. Melvin, Stephanie L. Laurinec, Benjamin S. Bassin

**Affiliations:** * University of Michigan, Department of Emergency Medicine, Ann Arbor, Michigan; † University of Michigan, Department of Emergency Medicine, Division of Critical Care, Ann Arbor, Michigan; ‡ Michigan Center for Integrative Research in Critical Care, University of Michigan, Ann Arbor, Michigan

## Abstract

**Introduction:**

Extubation of appropriate patients in the emergency department (ED) may be a strategy to avoid preventable or short-stay intensive care unit (ICU) admissions, and could allow for increased ventilator and ICU bed availability when demand outweighs supply. Extubation is infrequently performed in the ED, and a paucity of outcome data exists. Our objective was to descriptively analyze characteristics and outcomes of patients extubated in an ED-ICU setting.

**Methods:**

We conducted a retrospective observational study at an academic medical center in the United States. Adult ED patients extubated in the ED-ICU from 2015–2019 were retrospectively included and analyzed.

**Results:**

We identified 202 patients extubated in the ED-ICU; 42% were female and median age was 60.86 years. Locations of endotracheal intubation included the ED (68.3%), outside hospital ED (23.8%), and emergency medical services/prehospital (7.9%). Intubations were performed for airway protection (30.2%), esophagogastroduodenoscopy (27.7%), intoxication/ingestion (17.3%), respiratory failure (13.9%), seizure (7.4%), and other (3.5%). The median interval from ED arrival to extubation was 9.0 hours (interquartile range 6.2–13.6). One patient (0.5%) required unplanned re-intubation within 24 hours of extubation. The attending emergency physician (EP) at the time of extubation was not critical care fellowship trained in the majority (55.9%) of cases. Sixty patients (29.7%) were extubated compassionately; 80% of these died in the ED-ICU, 18.3% were admitted to medical-surgical units, and 1.7% were admitted to intensive care. Of the remaining patients extubated in the ED-ICU (n = 142, 70.3%), zero died in the ED-ICU, 61.3% were admitted to medical-surgical units, 9.9% were admitted to intensive care, and 28.2% were discharged home from the ED-ICU.

**Conclusion:**

Select ED patients were safely extubated in an ED-ICU by EPs. Only 7.4% required ICU admission, whereas if ED extubation had not been pursued most or all patients would have required ICU admission. Extubation by EPs of appropriately screened patients may help decrease ICU utilization, including when demand for ventilators or ICU beds is greater than supply. Future research is needed to prospectively study patients appropriate for ED extubation.

## INTRODUCTION

Endotracheal intubation is commonly performed in the emergency department (ED) and prehospital setting for respiratory failure or airway protection. Extubation is infrequently performed in the ED, possibly due to anticipated duration of underlying process requiring intubation, prompt transfer to the intensive care unit (ICU) when a bed is available, variability in clinician experience, and limited ability to monitor patients post-extubation. Many ICUs are facing increasingly strained capacity, resulting in ED boarding of critically ill patients.^1^ Demand for ventilators or ICU beds often outweighs supply, including during a pandemic. However, prolonged boarding of mechanically ventilated patients in the ED has been associated with worse outcomes.^2^

Extubation of appropriate patients in the ED may be a strategy to avoid preventable or short-stay ICU admissions. In addition, decreasing duration of mechanical ventilation likely reduces exposure to the harms associated with mechanical ventilation, which include barotrauma, lung injury, hypotension, gastrointestinal bleeding due to stress ulceration, ventilator-associated pneumonia, deconditioning, diaphragm weakness, and pneumothorax.^3^ Limited data regarding extubation of ED patients exists. A retrospective study of 50 trauma patients extubated in the ED suggested that, when appropriately screened for extubation readiness, 0% of patients required unplanned re-intubation, and a small subset was able to be discharged from the ED.^4^ Several additional patient populations have been identified as potentially appropriate for ED extubation,^5^,^6^ although published data supporting these practices is lacking.

A novel ED-ICU setting may provide an ideal environment for emergency physicians (EP) to safely extubate appropriate ED patients. The objective of this study was to descriptively analyze characteristics and outcomes of patients extubated in an ED-ICU.

## METHODS

### Study design and setting

This was a retrospective observational study conducted at a single, large, academic medical center in the United States with approximately 75,000 adult ED visits per year. The institutional review board at the University of Michigan reviewed and approved this study and determined it exempt from ongoing review. This study is presented in accordance with the Strengthening the Reporting of Observational Studies in Epidemiology (STROBE) statement.^7^

The Joyce and Don Massey Family Foundation Emergency Critical Care Center (EC3), an ED-ICU, was created in 2015 to provide comprehensive critical care for ED patients. The EC3 consists of five resuscitation bays and nine ICU-styled patient rooms adjoining the main ED. The EC3 is staffed by a separate team of EPs with or without critical care fellowship training, house staff, physician assistants, and ED nurses (with additional ICU training) who care for patients after initial management by the primary ED team.^8^

**Population Health Research Capsule**

What do we already know about this issue?

Extubation of appropriate patients in the emergency department (ED) may be a strategy to avoid preventable or short-stay intensive care unit (ICU) admissions, and could allow for improved ICU utilization.

What was the research question?

What were characteristics and outcomes of patients extubated in an ED-ICU setting?

What was the major finding of the study?

Extubations of 202 ED patients were included; 0.5% required re-intubation within 24 hours, and 7.4% required ICU admission.

How does this improve population health?

Extubation by emergency physicians of appropriately screened patients may help optimize ICU utilization by preventing avoidable ICU admissions.

### Selection of Participants

Adult ED patients extubated in EC3 from February 2015 through November 2019 were included and analyzed via retrospective review of the electronic health record (EHR). An EHR query identified patients with an order for “extubate patient” while in EC3. The authors manually reviewed all charts to ensure appropriateness for inclusion and data extraction. The date range, which determined the study size, was determined by the date EC3 opened (February 2015).

### Measurements and Outcomes

Age, gender, location of intubation, hours from ED arrival to extubation, ED disposition, unplanned re-intubation within 24 hours of extubation, and ED length of stay (LOS) (inclusive of time in both the main ED and ED-ICU) were collected from the EHR and analyzed. Individual case review was performed to identify indication for intubation, extubation for palliative purposes, and the attending physician at the time of extubation. The primary outcomes of interest were unplanned re-intubation rate and ED disposition. The study authors manually extracted data from the EHR, and a separate study author iteratively reviewed a subset of charts for quality assurance and to assure accuracy.

### Analysis

We performed a descriptive analysis of all patients meeting inclusion criteria. Separate subgroup analyses compared cohorts grouped by indication for intubation. Statistical analysis was performed using Microsoft Excel (Microsoft Corporation, Redmond, WA) and a TI-30X IIS (Texas Instruments, Dallas, TX) calculator. Analysis was conducted from December 2019 to March 2020.

## RESULTS

A total of 202 patients were identified and included for analysis; 85 (42%) were female and median age was 60.86 years (Table 1). Locations of endotracheal intubation included ED (n =138, 68.3%); outside hospital ED (n = 48, 23.8%), and emergency medical services/prehospital (n=16, 7.9%). Intubations were performed for indications of airway protection (n = 61, 30.2%); urgent esophagogastroduodenoscopy (EGD) (n = 56, 27.7%); intoxication/ingestion (n = 35, 17.3%); respiratory failure (n = 28, 13.9%); seizure (n=15, 7.4%); and other (n = 7, 3.5%).

The median interval from ED arrival to extubation was 9.0 hours (IQR 6.2–13.6). The median total ED LOS was 18.37 hours (interquartile range [IQR] 12.56–26.51) inclusive of time in both the ED and the ED-ICU. The median ED-ICU LOS was 14.8 hours (IQR 8.7–23.0). The overall rate of unplanned re-intubation within 24 hours of extubation in the ED-ICU was 0.5%. The attending emergency physician at the time of extubation was not critical care fellowship trained in 113 cases (55.9%).

We performed a subgroup analysis of patients who underwent palliative or compassionate extubation. This was defined as extubation with the expectation of imminent respiratory failure in order to relieve suffering associated with tracheal intubation and mechanical ventilation.^9^ Sixty patients underwent compassionate extubation in the ED-ICU, of whom 26 (43.3%) were female and median age was 76.63 years. In this group, the median interval from ED arrival to extubation was 7.0 hours (IQR 4.7–11.2), and the median total ED LOS was 14.0 hours (IQR 10.0–19.5). Forty-eight (80%) died in the ED-ICU, 11 (18.3%) were admitted to medical-surgical units, and one (1.7%) was admitted to intensive care. No patients were re-intubated within 24 hours of extubation. The attending EP was not critical care fellowship trained in 32 cases (53.3%).

Of patients *not* extubated compassionately (n =142), zero died in the ED-ICU. Eighty-seven (61.3%) were admitted to medical-surgical units, 14 (9.9%) were admitted to intensive care, 40 (28.2%) were discharged from the ED-ICU, and one (0.7%) was transferred to another facility. The median time interval from ED arrival to time of extubation was 9.9 hours (IQR 7.0–15.0). The rate of unplanned re-intubation within 24 hours of extubation in the ED-ICU was 0.7%. The attending EP was not critical care fellowship trained in 81 cases (57.0%).

## DISCUSSION

This study suggests select ED patients can be safely extubated in an ED-ICU by EPs. This practice appears associated with reduced short-stay ICU admissions. Optimal utilization and allocation of ICU resources, including ventilators, is essential when ICU capacity is under strain and demand outweighs supply. ED extubation of appropriately selected patients in appropriately monitored settings is one strategy to help decrease ICU utilization.

There is a paucity of data regarding extubation of ED patients. To our knowledge, the only existing case series of patients extubated in the ED included only patients intubated in the setting of trauma.^4^ In our study, zero of the observed cohort were intubated in the setting of trauma, although several other patient populations were extubated in the ED. These included patients requiring intubation for airway protection in the setting of transient central nervous system depression, need for urgent EGD, acute intoxication, and seizure. These are consistent with groups previously identified as appropriate for ED extubation.^6^ As a spectrum of illness severity exists within these populations, it is imperative the underlying process requiring intubation has resolved prior to consideration of extubation. Patients pursuing palliative care may also benefit from ED extubation, and compassionate extubation has the benefit of limiting patient and family suffering and facilitating end-of-life care when appropriate resources exist.

One patient required unplanned re-intubation within 24 hours of extubation during the study period. The patient was initially intubated for airway protection in the setting of agitated delirium requiring sedation to facilitate diagnostics, therapeutics, and patient and staff safety. The patient was extubated in the ED-ICU by an EP with critical care fellowship training, and was re-intubated about nine hours later for acute hypoxic respiratory failure and ongoing agitated delirium requiring sedation for patient and staff safety. He was extubated in the inpatient ICU two days later, and discharged five days later with no appreciable complications of either intubation or mechanical ventilation.

The observed rate of extubation failure was 0.5%. This is lower than previously published rates of 10 – 20% for all ICU patients,^11^–^13^ although these rates were derived from more heterogeneous groups of ICU patients with longer durations of mechanical ventilation and protracted illness. These findings suggest ED extubation is safe in appropriately screened patients at low risk for extubation failure. No established criteria for extubation readiness were used in our patient population, although an extubation readiness protocol and future prospective study of this practice may be beneficial.

Management of rapidly reversible critical illness, including extubation of appropriately screened patients, in an ED-ICU may help alleviate strain facing many inpatient ICUs. Preventing short-stay ICU admissions is one strategy to optimize ICU bed allocation for patients decompensating on wards, ICU-to-ICU transfers, and patients with more prolonged critical care needs.^8^,^14^–^16^ In our study, only 7.4% of the observed patient population required an inpatient ICU admission after receiving care in the ED-ICU, and 43.6% did not require hospitalization. In the absence of ED extubation or an ED-ICU, we hypothesize that the vast majority (perhaps even all) of these patients would have been admitted to an inpatient ICU only to quickly undergo extubation and transfer. EP-performed extubation of appropriately selected patients may help reduce short-stay ICU admissions and optimize inpatient ICU bed utilization.

Endotracheal intubation and mechanical ventilation have been associated with a number of adverse events. These include barotrauma, laryngeal injury, lung injury, hypotension, gastrointestinal bleeding due to stress ulceration, ventilator associated pneumonia, deconditioning, diaphragm weakness, and pneumothorax.^3^,^17^–^23^ Extubation should be performed as soon as appropriate to mitigate these risks. Physical location should not preclude extubation when indicated and appropriate resources are present, and the ED (or ED-ICU) is often the location where extubation is first appropriate. This study helps demonstrate the safety and feasibility of extubation in this environment.

## LIMITATIONS

The retrospective observational nature of this study limits interpretation of results to association rather than causation. This study was conducted at a single, academic medical center in the United States, in a unique setting (ED-ICU) with a low patient-to-nurse ratio of 2:1.^10^ For the EP, the evaluation of a patient, decision to extubate, performance of extubation, and monitoring post-extubation presents a task that can require time and effort, while competing demands for time and effort are constantly present. An ED-ICU may help mitigate this challenge. Thus, the generalizability of these results to many EDs may be limited. The sample size in each subgroup is relatively small, due to the relative infrequency of ED extubation. No specific criteria existed for determination of patients’ readiness for ED extubation, and patients were determined suitable for extubation at the attending physician’s discretion.

Nearly half (44.1%) of extubations were performed by EPs with critical care fellowship training, who may have possessed more familiarity with liberation from mechanical ventilation than non-fellowship trained EPs. Still, the majority of extubations were performed by EPs without critical care fellowship training, suggesting critical care fellowship training is likely not necessary to safely make this decision in many cases. However, we did not compare extubation experience or success between fellowship-trained and non-fellowship trained physicians. A larger proportion of our study subjects had been intubated prior to transfer to our hospital (23.8%), or for time-limited procedures (EGD; 27.7%) than would be typical for a community hospital. Data was manually extracted from the EHR by study authors who were not blinded to the study hypothesis, and was directly input to a spreadsheet, although no formal standardized data sheet was used otherwise.

## CONCLUSION

This study demonstrates that select ED patients can be safely extubated in an ED-ICU by EPs. Only 7.4% required ICU admission; whereas if ED extubation had not been not pursued most or all patients would have required ICU admission. Management of rapidly reversible critical illness, including extubation of appropriately screened patients, in an ED-ICU may help optimize ICU utilization by preventing avoidable short-stay ICU admissions, including when demand for ventilators or ICU beds is greater than supply. With increasing ED boarding of critically ill patients, ED extubation may contribute to more effective allocation of inpatient critical care resources. Future research is needed to prospectively study patients appropriate for ED extubation, and to assess the impact of an ED-ICU on additional critically ill patient populations.

## Figures and Tables

**Table 1:** Characteristics of patients extubated in emergency department-based intensive care unit, by indication for intubation.

	All extubations, n = 202	Compassionate extubations*, n = 60	All other extubations, n = 142	Intoxication/ingestion, n = 34	Seizure, n = 15	Esophago-gastroduo-denoscopy, n = 55	Airway protection/depressed mental status, n = 21	Respiratory failure, n = 14	Other, n= 3
Age, median (years)	60.86	76.63	51.48	36.59	49.7	55.2	62.2	69.1	69.7
Gender, female (%)	42.1	43.3	41.5	35.3	33.3	38.2	42.9	71.4	66.7
Location of intubation (%)									
ED	68.3	55.0	73.9	88.2	46.7	92.7	47.6	35.7	66.7
Prehospital/EMS	7.9	16.7	4.2	8.8	0	1.8	4.8	7.1	0
Outside hospital ED	23.8	28.3	21.8	2.9	53.3	5.5	47.6	57.1	33.3
Hours from ED arrival to extubation, median (IQR)	9.0 (6.2–13.6)	7.0 (4.7–11.2)	9.9 (7.0–15.0)	9.5 (7.1–16.1)	7.1 (5.6–11.7)	10.2 (7.4–13.9)	10.1 (6.7–12.2)	9.3 (6.5–15.6)	20.3 (18.1–27.4)
ED disposition, n (%)									
Admission to intensive care unit	15 (7.4)	1 (1.7)	14 (9.9)	2 (5.9)	0 (0)	4 (7.3)	5 (23.8)	3 (21.4)	0 (0)
Admission to general ward	98 (48.5)	11 (18.3)	87 (61.3)	15 (44.1)	14 (93.3)	34 (61.8)	13 (61.9)	8 (57.1)	3 (100)
Discharge	40 (19.8)	0 (0)	40 (28.2)	17 (50)	1 (6.7)	16 (29.1)	3 (14.3)	3 (21.4)	0 (0)
Deceased	48 (23.8)	48 (80)	0 (0)	0 (0)	0 (0)	0 (0)	0 (0)	0 (0)	0 (0)
Transfer to another facility	1 (0.5)	0 (0)	1 (0.7)	0 (0)	0 (0)	1 (1.8)	0 (0)	0 (0)	0 (0)
Unplanned re-intubation within 24 hours, n (%)	1 (0.5)	0 (0)	1 (0.7)	0 (0)	0 (0)	0 (0)	1 (4.8)	0 (0)	0 (0)
Median ED length of stay, hours	18.37 (12.56–26.51)	14.0 (10.0–19.5)	20.5 (13.8–27.3)	19.8 (12.8–29.0)	23.6 (15.3–26.3)	19.3 (13.4–26.4)	20.2 (16.0–25.2)	26.4 (17.7–29.8)	36.9 (32.7–39.1)

*ED*, emergency department; *EMS*, emergency medical services; *IQR*, interquartile range.

* Compassionate extubation: performed with expectation patient would not adequately maintain respiratory status, and to limit patient and family suffering.
